# Investigation into the Co-Phase Detection Methodology for Segmented Plane Mirrors Utilizing Grazing Incidence Interferometry

**DOI:** 10.3390/s24030904

**Published:** 2024-01-30

**Authors:** Rengcong Liu, Jiang Guo, Yibo Li

**Affiliations:** 1 Changchun Institute of Optics, Fine Mechanics and Physics, Chinese Academy of Sciences, Changchun 130033, China; liurengcong22@mails.ucas.ac.cn (R.L.); liyibo221@mails.ucas.ac.cn (Y.L.); 2 University of Chinese Academy of Sciences, Beijing 100049, China

**Keywords:** co-phase detection, grazing incidence, interference detection, segmented plane mirrors, piston error, 2π ambiguity

## Abstract

Segmented plane mirrors constitute a crucial component in the self-aligned detection process for large-aperture space optical imaging systems. Surface shape errors inherent in segmented plane mirrors primarily manifest as tilt errors and piston errors between sub-mirrors. While the detection and adjustment techniques for tilt errors are well-established, addressing piston errors poses a more formidable challenge. This study introduces a novel approach to achieve long-range, high-precision, and efficient co-phase detection of segmented plane mirrors by proposing a segmented plane mirror shape detection method based on grazing incidence interferometry. This method serves to broaden the detection range of piston errors, mitigate the issue of the 2π ambiguity resulting from piston errors in co-phase detection, and extend the detection capabilities of the interferometer. By manipulating the incident angle of the interferometer, both rough and precise adjustments of the segmented plane mirrors can be effectively executed.

## 1. Introduction

**Figure 1:**
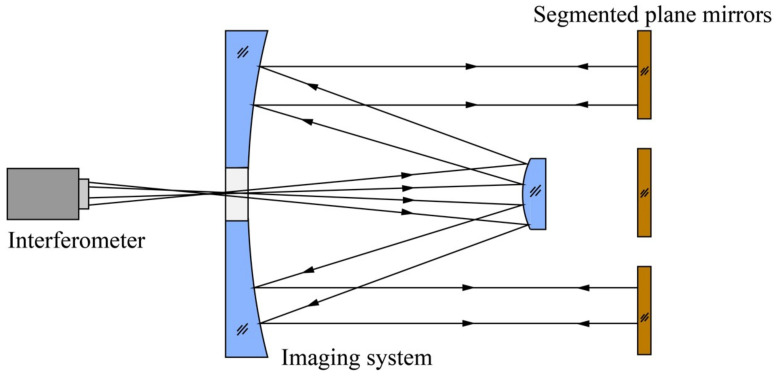
Schematic of self-aligned detection of a large-aperture optical imaging system.

In customary practice, self-aligned detection serves as a method for scrutinizing spatial optical imaging systems. The conceptual framework of self-aligned detection is depicted in Figure 1. In this schema, the interferometer releases spherical waves precisely at the focal point of the imaging system. Subsequently, these waves transform into parallel light upon traversing the optical system, ultimately retracing their path along the original optical trajectory upon encountering reflection by the plane mirror. The interferometer then collects the wavefront information of the system, providing a comprehensive insight into its optical characteristics. Notably, as the aperture of the space optical imaging system expands, a commensurate increase in the aperture of the plane mirror utilized for self-alignment detection becomes imperative.

In the United States, the self-aligned detection of the JWST optical system is achieved by moving and adjusting three 1.5 m aperture mirrors, and the co-phase adjustment of the primary mirror is achieved by using a multi-wavelength interferometer [1], which requires a variety of measurement equipment (including special customized equipment), and the completion of the detection is very costly and time-consuming. If the large-aperture space telescope is subjected to high-precision, high-efficiency, and low-cost self-aligned detection, it can be considered to break through the aperture limitation of the traditional single-plane mirror and increase the equivalent aperture of the plane mirror by using the method of sub-mirror segmentation. At present, the grazing incidence interferometric detection method of a single-plane mirror is mature, and the detection method has reached high accuracy [2,3,4]. However, the use of this method to complete the detection of large-aperture segmented plane mirrors is still a difficult problem, not only because large-aperture segmented plane mirrors are less widely used (to overcome the technical bottleneck of a traditional single-plane mirror with a diameter of less than 8 m), but also because piston errors in segmented plane mirrors are more difficult to detect than in single-plane mirrors. To uphold the facial shape precision of these segmented plane mirrors, it becomes imperative to identify and rectify the piston and tilt errors among the sub-mirrors. While the tilt error detection technique has achieved a certain degree of maturity, the predicament surrounding piston error detection remains unresolved, primarily attributable to issues such as 2π blurring and related challenges [5].

Various methodologies are available for the detection of segmented plane mirrors, including broad- and narrow-band Shack–Hartmann detection [6], dispersion fringe method [7], pentaprism scanning detection [8], dual-wavelength co-phase detection [9], and interference detection method [10]. Refer to Table 1 for an elucidation of their respective merits and demerits.

Based on the table, the interference detection method approach manifests noteworthy precision and efficiency in detection. The enhancement of surface shape adjustment quality for segmented plane mirrors becomes feasible upon resolution of issues associated with limited detection range and 2π ambiguity. In response to this challenge, a novel co-phase detection methodology for segmented plane mirrors, grounded in the principles of grazing incidence interferometric detection, is introduced in this paper. The proposed method involves augmenting the incident angle of the interferometer to broaden the individual detection range of segmented plane mirrors. Concurrently, a slight tilt is introduced to the standard plane mirror to mitigate the complexity in detecting piston errors arising from the 2π ambiguity. Moreover, through manipulation of the incident angle of the interferometer, both rough and precise adjustments of the segmented plane mirrors can be accomplished. This innovative approach addresses the identified limitations, thereby offering a comprehensive solution for improving the efficacy of surface shape adjustment in segmented plane mirrors.

## 2. Principle of the Grazing Incidence Interferometric Detection Method

### 2.1. Basic Principle and Piston Detection Range

**Figure 2:**
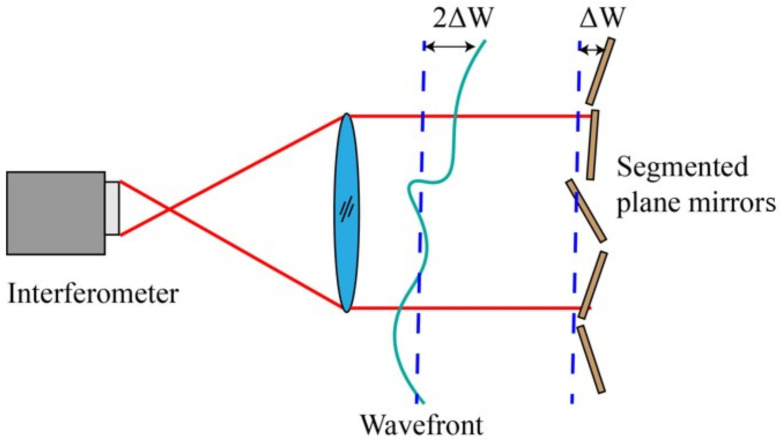
Principle of interference detection.

The illustration of interference detection principles is depicted in Figure 2. The homogenous light beam emanating from the interferometer aligns with the segmented plane mirrors under scrutiny. Subsequently, it undergoes reflection and retraces its original optical trajectory, culminating in the generation of an interferogram upon retrieval by the interferometer. The segmented plane mirrors typically display a patter of interference fringes when examined for interference. Any tilt error results in alterations to the direction and density of these fringes, whereas a piston error leads to periodic changes in the overall phase of the interference images, specifically the translation of fringes. Correcting tilt errors involves adjusting the direction and density of interference fringes, while assessing piston errors relies on examining the continuity of fringes between adjacent sub-mirrors. Notably, a change of 0.5λ in piston errors corresponds to a single-period shift in the interference fringe, posing a challenge in accurately detecting piston errors exceeding 0.5λ due to a 2π ambiguity.

**Figure 3:**
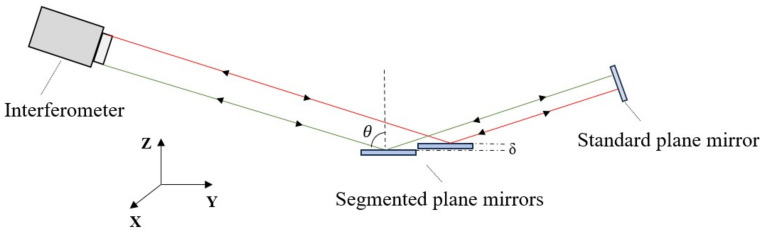
Principle of grazing incidence interferometric detection method.

**Figure 4:**
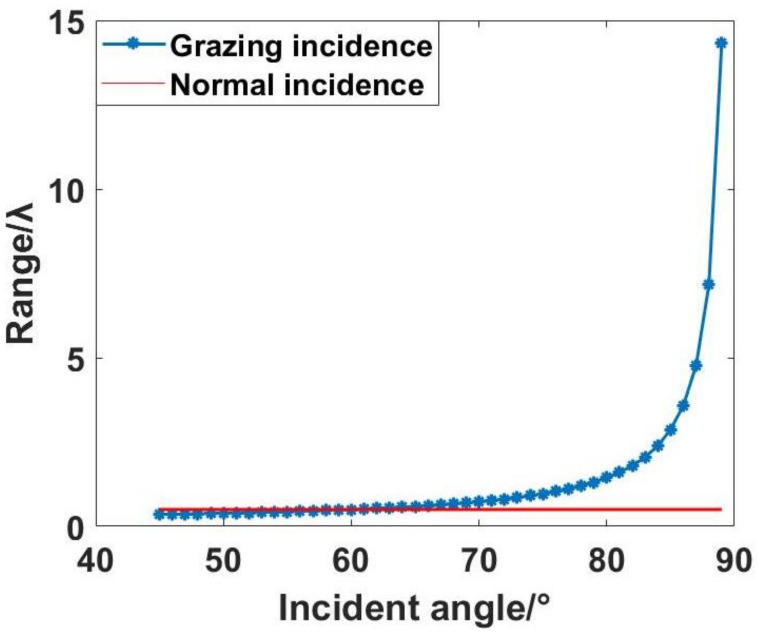
Relationship between piston error range and incident angle.

Figure 3 depicts the principle of grazing incidence detection. In the scenario where the interferometer is inclined at an angle θ during incidence, the emitted light beam from the interferometer illuminates the segmented plane mirrors. Subsequently, upon reflection by the standard plane mirror, the light retraces its original path. This orchestrated interplay facilitates the derivation of the intensity distribution $I_{\theta }\left(x,y\right)$ pertaining to the interference fringes.

(1) $$I_{\theta }\left(x,y\right)=I_{0}-I_{0}cos\left\{\frac{2\pi }{\lambda }\cdot 4\cos\theta \left[a_{i}x+b_{i}y+c_{i}\right]\right\}$$

where $a_{i}$, $b_{i}$, and $c_{i}$ correspond to the *x*-tilt, *y*-tilt, and piston error coefficients of the *i*-th sub-mirror, respectively. At this juncture, the modulation of the interference fringes undergoes precise translation by a single cycle with alterations in the parameter $c_{i}$, specifically when it varies by $\frac{\lambda }{8\cos\theta }$. In essence, the introduction of grazing incidence allows for an extension of the discernible range for piston errors from 0.5λ to $\frac{\lambda }{8\cos\theta }$. The correlation between the angle of grazing incidence and the expanded detection range of piston errors is illustrated in Figure 4.

As depicted in the graphical representation, beyond an incident angle surpassing 60°, the detection range associated with grazing incidence surpasses that of interferometry. Furthermore, there is an accelerated augmentation in the detection range as the angle of incidence intensifies. Specifically, at an incident angle of 85°, the theoretical detection range attains 2.868λ, with a notable escalation in its rate of increase corresponding to higher incident angles. Notably, at an incident angle of 89°, the theoretical detection range achieves a remarkable extent of 14.32λ. If the incident angle continues to increase, less incident light will be reflected, and when the incident angle increases to 90°, there will be no reflected light.

### 2.2. Scope and Accuracy of Detection

The utilization of grazing incidence detection proves to be efficacious in broadening the scope of incident direction detection. In the context of interferometric detection with an aperture size denoted as *D*, the incident angle denoted as θ facilitates an expansion of the incident direction detection scope to $\frac{D}{\cos\theta }$.

Generally, segmented plane mirrors characterized by an overall RMS face shape superior to λ/20 can satisfy the specified criteria for their application. Within the context of the grazing incidence detection method, a decline in detection accuracy is observed as a consequence of heightened incident angle, the incorporation of standard plane mirror, and an escalation in the quantity of reflections [11]. The wave aberration of the system is articulable as:

(2) W=4δcosθ+2ε

In the aforementioned equation, δ signifies the surface shape discrepancy of the segmented plane mirrors subject to detection; θ denotes the incident angle of grazing incidence; ε characterizes the surface discrepancy of the standard plane mirror. Given that the RMS accuracy of the wavefront detected by the interferometer is denoted as *r*, the corresponding RMS detection accuracy $\delta_{t}$ of the segmented plane mirrors is determined as follows:

(3) $$\delta_{t}=\frac{\sqrt{r^{2}+{\left(2\varepsilon \right)}^{2}}}{4\cos\theta }$$

**Figure 5:**
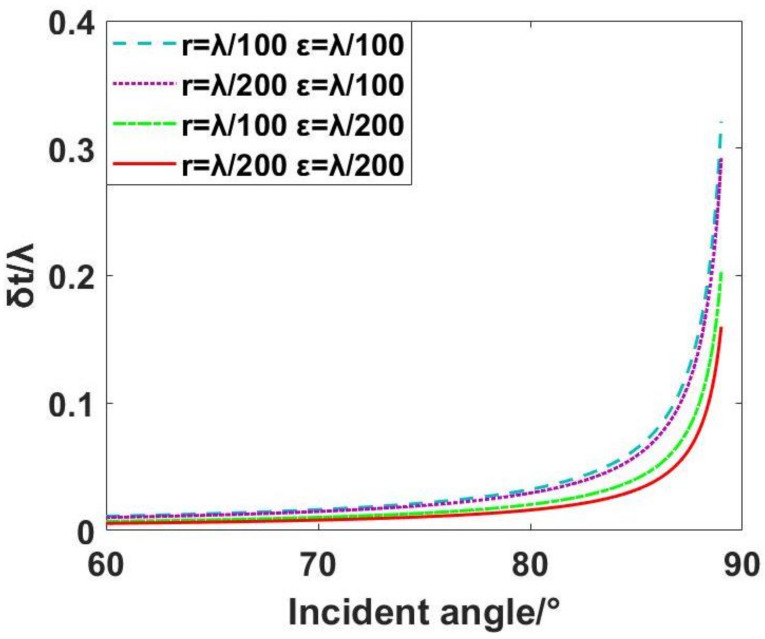
Relationship between detectable RMS accuracy and incident angle.

Since the previous range analysis shows that the range is larger than the interference detection method when the incident angle is greater than 60°, this research method is meaningful. Therefore, the incident angle should be increased from 60° when using this method. The graphical representation in Figure 5 illustrates the dependency of $\delta_{t}$ on the incident angle, considering values of r at λ/100 and λ/200, as well as ε at λ/100 and λ/200.

The graphical representation illustrates that, at an incident angle of 85°, optimal detection accuracy exceeding λ/20 can be achieved through the selection of an interferometer characterized by high detection accuracy and a standard plane mirror with precise surface shape characteristics. Concurrently, this configuration results in an expanded single detection range of the interferometer and a larger range for detecting piston errors, facilitating the rough adjustment of the segmented plane mirrors. Conversely, at an incident angle of 75°, superior detection accuracy surpassing λ/50 is attainable, albeit with a narrower measurement range. This configuration is conducive to the precise adjustment of the segmented plane mirrors.

**Figure 6:**
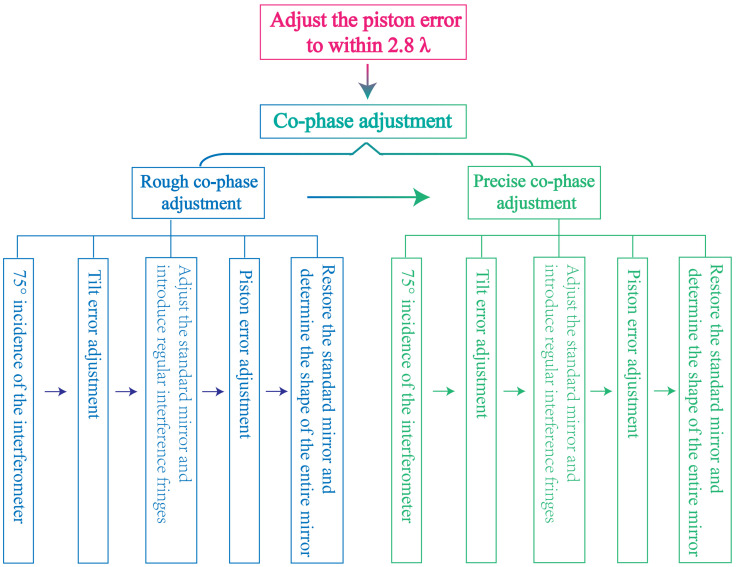
Co-phase adjustment process of segmented plane mirrors based on grazing incidence interferometric detection method.

Moreover, the efficacy of the interferometer in detecting a segmented plane mirror surface is contingent upon both its detection accuracy and the surface precision of the standard plane mirror. Significantly, the impact of the standard plane mirror’s surface accuracy on the overall surface detection process is more pronounced. Consequently, in the practical application of segmented plane mirrors through swept incidence detection in engineering, it is imperative to enhance the surface accuracy of standard plane mirror to the greatest extent possible. This enhancement facilitates the achievement of heightened precision in detection. The co-phase adjustment process of segmented plane mirrors, employing the grazing incidence interferometric detection method, is illustrated using Figure 6.

## 3. Simulation Analysis

### 3.1. Interference Fringe Processing Method

The analytical techniques employed for interference fringe images can be broadly categorized into two main groups: the full grayscale method and the fringe centerline method. Within the full grayscale method, noteworthy approaches encompass the Phase Shifting Method (PSM) and the Fourier Transform Method (FTM) [12]. The PSM necessitates a high degree of environmental stability, while the FTM encounters challenges in mitigating the impact of noise. In contrast, the fringe centerline method constitutes an optical interferometric image analysis approach that leverages the grayscale analysis of fringes. This methodology mandates the refinement of fringes before centerline extraction, thereby yielding a substantial enhancement in measurement accuracy.

The enhancement of the interference fringe map is conducted through the utilization of binary graphs. Binarization algorithms applied to interferometric streak maps may derive from either the direct binarization of the grayscale graph [13] or employ edge detection techniques [14]. It is crucial to note that the refinement algorithm exhibits a heightened sensitivity to both white noise and edge irregularities present within the fringes. Consequently, it necessitates the implementation of smoothing and de-noising procedures for optimal performance.

**Figure 7:**
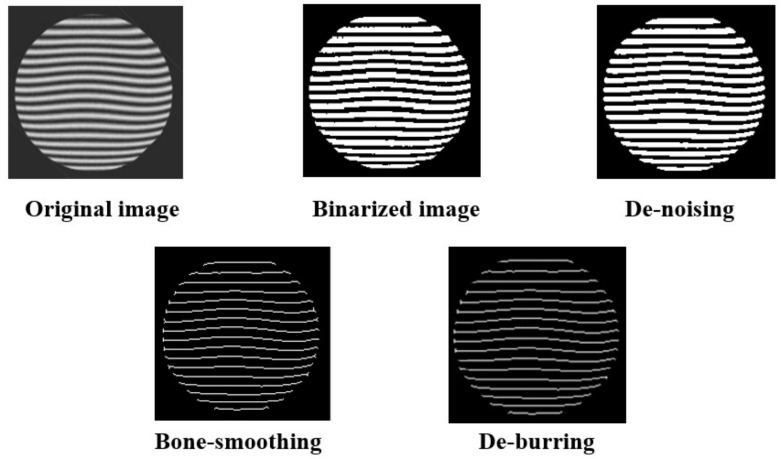
Schematic diagram of interference fringe image processing.

In the present study, we opted for a series of sequential processing steps for the manipulation of interference fringe images. Specifically, the chosen processing methodology involves the successive application of grayscale conversion, binary image conversion, high-pass filtering for noise mitigation, bone-smoothing, and de-burring techniques, as illustrated in Figure 7.

### 3.2. Simulation of Grazing Incidence Interferometric Detection

**Figure 8:**
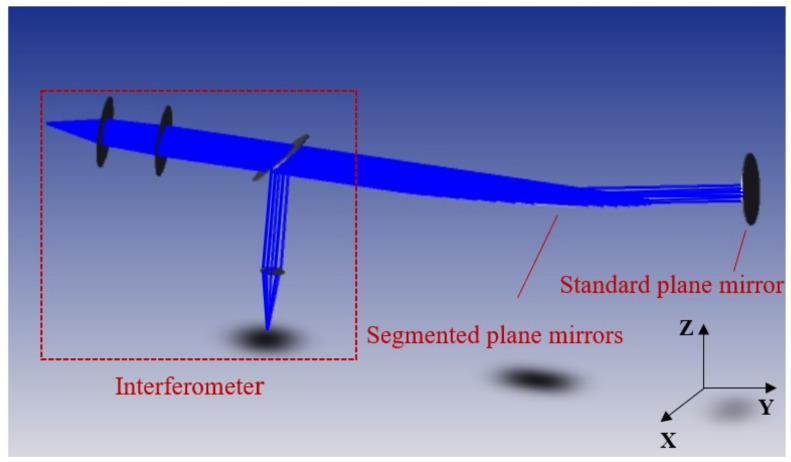
Schematic diagram of grazing incidence interferometic detection in simulation.

The grazing incidence interferometric detection is simulated. Firstly, the interferometer is constructed by simulation. The beam emitted by the interferometer is illuminated on the segmented plane mirrors and then returned by the standard plane mirror, as illustrated in Figure 8.

**Figure 9:**
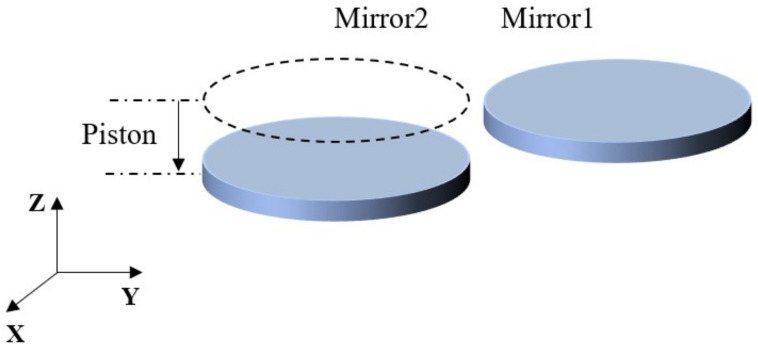
Schematic diagram of piston errors.

In the simulation, a configuration employing two circular plane mirrors as segmented plane mirrors is employed for the purpose of accomplishing grazing incidence interferometric detection. The segmented nature of the plane mirrors induces piston errors through the Z-directional translation of Mirror2, as illustrated in Figure 9.

**Figure 10:**
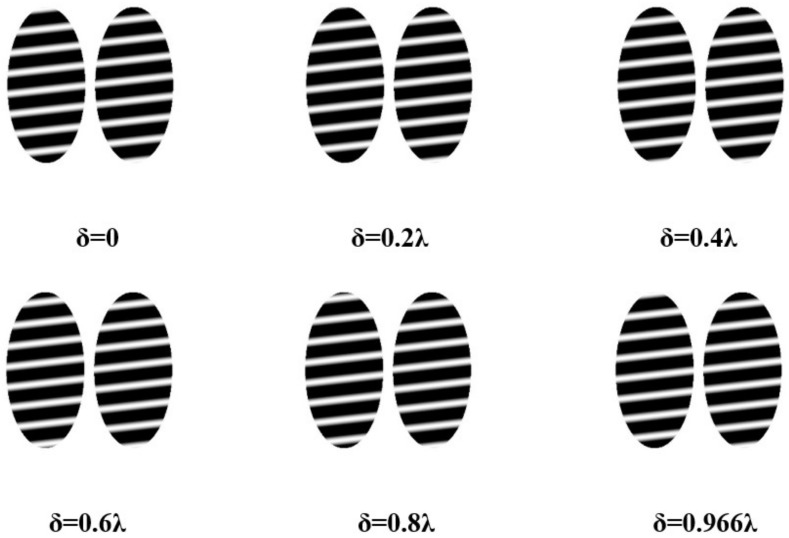
Schematic diagram of grazing incidence interference fringes changing with piston errors at an incident angle of 75°.

Within the simulation, the standard plane mirror undergoes adjustments, incorporating a subtle tilt to induce transverse interference fringes. The grazing incidence interference detection with an incident angle of 75° is simulated and analyzed. The ensuing analysis reveals a discernible alteration in interference fringes corresponding to the piston error δ, as illustrated in Figure 10. Notably, the leftward positioning of the interference fringes pertaining to Mirror2 is observed in the graphical representation.

The analysis of the interference fringes exhibited by Mirror2 in the depicted figure reveals a variation in piston errors ranging from 0 to 0.966λ. Additionally, the interference fringe pattern undergoes a single cycle alteration. In other words, the permissible range for the piston errors of the segmented plane mirrors, when employed for interferometric detection at a grazing incident angle of 75°, is determined to be 0.966λ.

**Figure 11:**
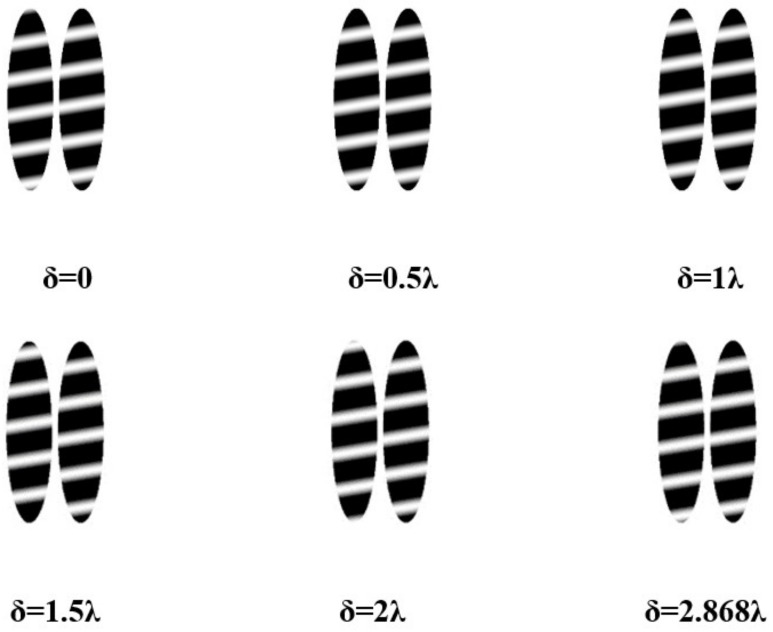
Schematic diagram of grazing incidence interference fringes changing with piston errors at an incident angle of 85°.

Within the simulation, the standard plane mirror undergoes adjustments to incorporate a specified degree of tilt aimed at generating interference fringes. The analytical examination of interference detection, conducted under a sweeping incident angle set at 85°, yields interference fringes characterized by their dependence on the piston error δ. This observation is illustrated in Figure 11, wherein the left portion depicts the interference fringes associated with Mirror2.

The analysis of the interference fringes observed in Mirror2, as depicted in the aforementioned figure, reveals that the piston errors ranging from 0 to 2.868λ. Additionally, it is observed that the interference fringe configuration undergoes a single cycle alteration. Specifically, the segmented plane mirrors, characterized by an incident angle of 85°, manifest piston errors within the range of 2.868λ.

**Figure 12:**
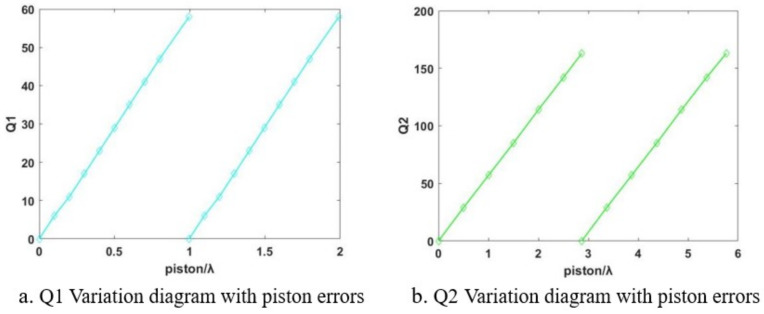
Schematic representation of the changes in Q1 and Q2 due to piston errors.

Q is defined as the ratio between the travel distance of interference fringes and the spacing of fringes. Simulations on the grazing incidence interferometric detection of the ratio between the travel distance of interference fringes and fringe spacing were performed, denoted as Q1 (incident angle of 75°) and Q2 (incident angle of 75°), for segmented plane mirrors. The relationship between Q1 and the piston errors and the relationship between Q2 and the piston errors is illustrated in Figure 12.

Figure 12 illustrates that the ratio of the travel distance of interference fringes to the fringe spacing (Q1, Q2), along with the associated piston errors, exhibits an approximate proportionality. Consequently, the interferogram acquired through grazing incidence interferometric detection proves instrumental in gauging the piston errors of the segmented plane mirrors. This is accomplished by scrutinizing and processing the ratio of the travel distance of interference fringes to the fringe spacing Q within the interferogram cycle. Additionally, the incident angle of the interferometer is modifiable within the range of 85° to 75°. This adjustment facilitates the comprehensive assessment of the piston errors of the segmented plane mirrors across a broad spectrum, subsequently enabling the refinement of the piston errors from rough to precise adjustments.

In summary, compared with the interference detection method, which has a measuring range of only 0.5λ, the grazing incidence interferometric detection method significantly expands the measuring range. Compared with the dual-wavelength co-phase detection, this method breaks through the aperture limitation of the interferometer and obviously expands the scope of detection. And this method only needs to adjust the incident angle of the interferometer, which is much more efficient than broad- and narrow-band Shack–Hartmann detection and pentaprism scanning detection.

## 4. Experiment Analyses

**Figure 13:**
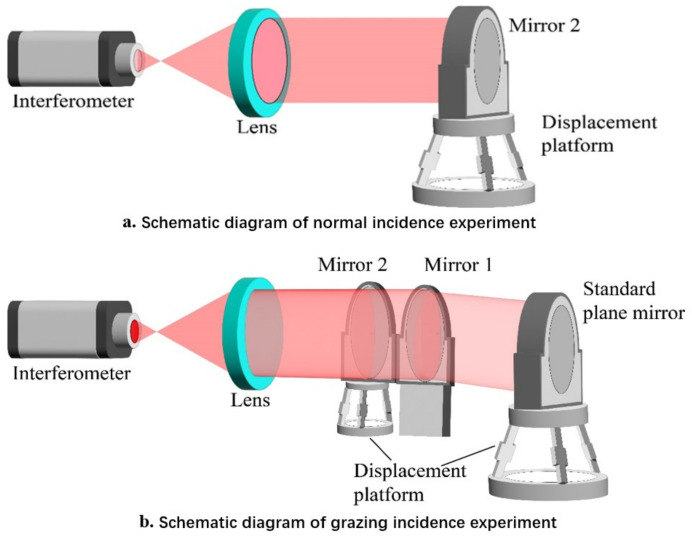
Schematic diagram of experimental arrangement and devices.

In this experimental investigation, an interferometer featuring an operational wavelength denoted as λ = 632.8 nm was employed (this wavelength of light is relatively stable and suitable for interferometric applications that require precise wavelength control). Mirror1 and Mirror2 are plane mirrors characterized by a shape accuracy of 1/20λ and possess an aperture size of 100 mm. The standard plane mirror utilized in the experiment exhibits a shape accuracy of 1/80λ and is equipped with an aperture measuring 150 mm. The actuation mechanism for Mirror2 is facilitated by the H-811.I2 hexapod displacement platform, developed by the PI Company. This hexapod platform exhibits a minimum displacement of 100 nm in both the X and Y directions, a minimum displacement of 80 nm in the Z direction, and boasts a design resolution for the actuator set at 5 nm. The detailed schematic representation of the experimental arrangement and the employed devices is presented in Figure 13.

**Figure 14:**
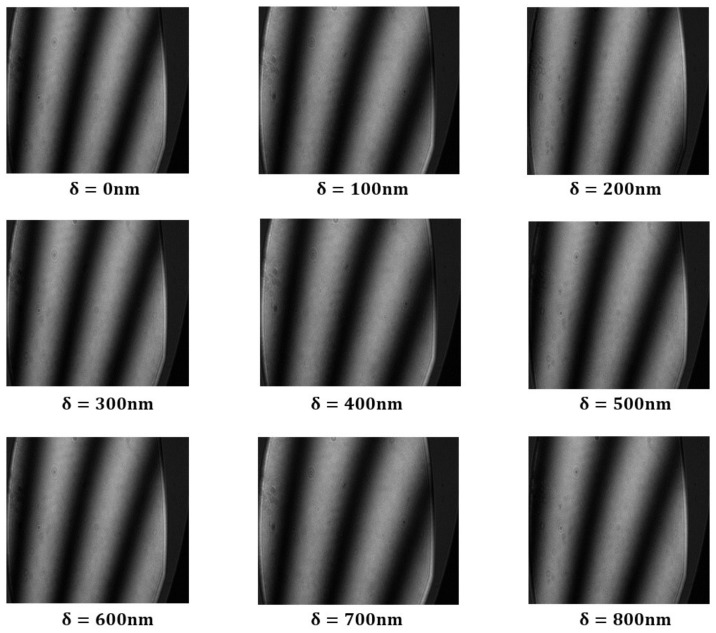
Variation diagram of interference fringes in normal incidence experiment.

The initial positioning of Mirror2 involves its placement before the interferometer, with the optical path exhibiting normal incidence, as illustrated in Figure 13a. In this experiment, the distance between the interferometer and Mirror2 is 500 mm. The employment of normal incidence within the interferometer serves as a means of detection, where a minute tilt of Mirror2 was deliberately induced to generate interference fringes. Subsequently, the resulting interference patterns were meticulously documented. The piston error values of periodic variation in interference fringes in the previous simulation and the minimum displacement of the hexapod displacement platform are considered, the translational discrepancy, denoted as δ, was systematically incremented by 100 nm increments, and the corresponding interference patterns were meticulously captured, as depicted in Figure 14.

The analysis of the interferogram depicted in the preceding figure indicates a fluctuation in piston errors within the range of 0 to 300 nanometers (approximately 0.5λ). Moreover, the interference fringe pattern undergoes a single cycle alteration. This signifies that the effective normal incidence interferometric detection range is restricted to 0.5λ. This observation aligns with the anticipated theoretical limits of normal incidence detection.

**Figure 15:**
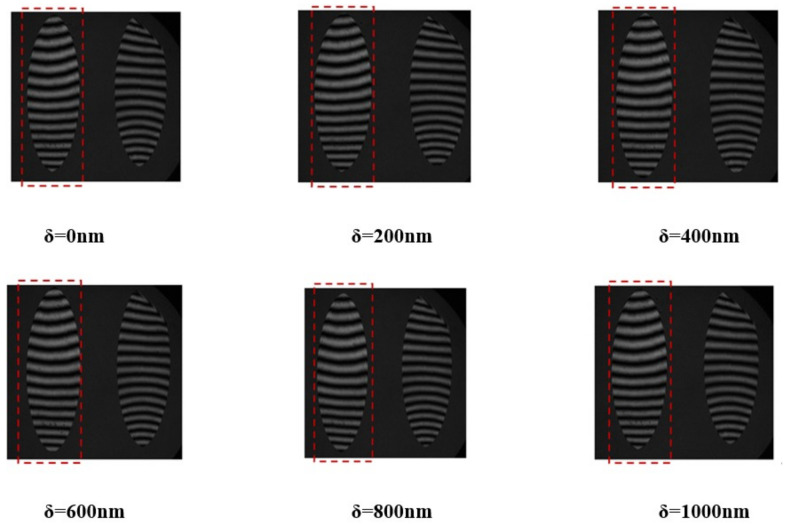
Variation diagram of interference fringes in 75° incidence experiment.

Following the synchronization of Mirror1 and Mirror2 to achieve a coherent phase, the optical trajectory for the detection of grazing incidence is delineated in Figure 13b. The distance from the interferometer to Mirror2 is still 500 mm, while the distance from the standard plane mirror to Mirror1 and Mirror2 is 900 mm. Establishing the incident angle for detection at 75°, the standard plane mirror was fine-tuned to induce a slight tilt, generating interference fringes. Mirror1 was maintained in a fixed position, while Mirror2 was incrementally displaced in 200 nm increments to induce piston errors. Subsequently, the resultant interference pattern was documented, as illustrated in Figure 15.

The interference pattern generated by Mirror 2 in the depicted illustration was analyzed to derive the piston errors within the range of 0 to 600 nm (0.95λ). Consequently, the interference fringe pattern underwent a singular cycle alteration. Specifically, the experimental interferometer, operating at an incident angle of 75°, effectively discerns the segmented plane mirrors, while the piston error detection range is restricted to 0.95λ. This observation aligns closely with both the theoretical and simulated projections for the piston error detection range, which is established at 0.966λ.

**Figure 16:**
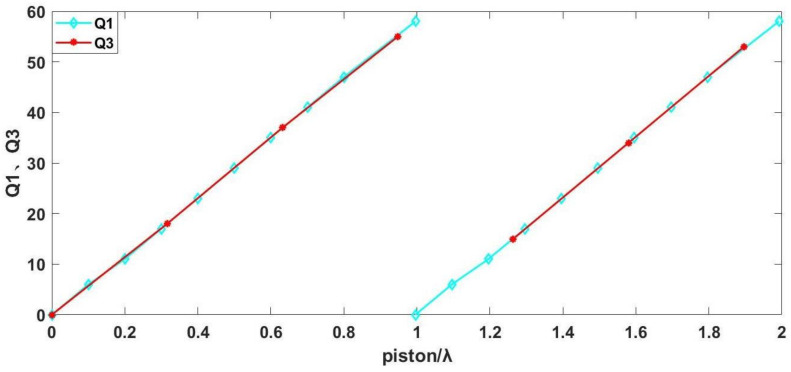
Schematic diagram of the relationship between Q1, Q3, and piston errors at 75° incidence.

The ratio between the travel distance of interference fringes to the spacing of fringes in the 75° incidence experiment is defined as Q3, and the correlation between Q3 and the piston errors was analyzed in contrast to the relationship observed between Q1 and the piston errors in the preceding 75° incidence simulation, as depicted in Figure 16.

The depicted diagram illustrates a notable similarity in the association between Q3 and piston errors observed in the 75° incidence experiment and the analogous relationship between Q1 and piston errors.

**Figure 17:**
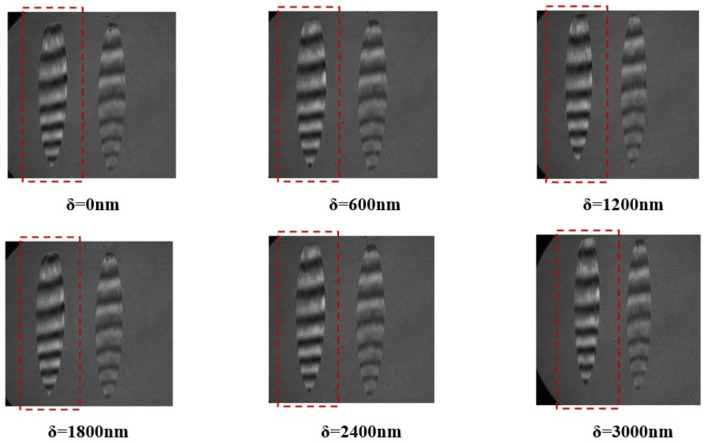
Variation diagram of interference fringes in 85° incidence experiment.

Upon aligning Mirror1 and Mirror2 to achieve co-phase restoration, the incident angle for detection was established at 85°. The standard plane mirror was then calibrated to induce a subtle inclination, thereby generating interference fringes. While Mirror1 remained fixed, Mirror2 was systematically manipulated to introduce piston errors in increments of 600 nm. Subsequently, the resultant interference pattern was documented, as illustrated in Figure 17.

**Figure 18:**
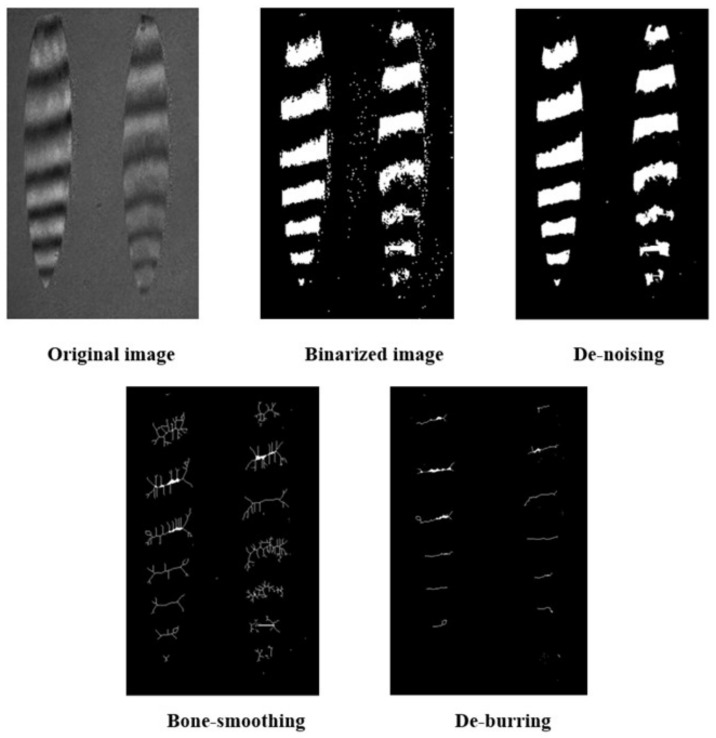
Diagram of interference fringe image processing.

From the above figure, it can be seen that when the grazing incident angle is large, the wavefront error of the mirror will significantly affect the interference fringes, but the center of the fringes can still be accurately extracted by processing the interference fringe pattern, as shown in Figure 18 (with δ = 0 nm as an example).

The interference pattern emanating from Mirror2 was subjected to meticulous processing to ascertain the piston errors within the discernible spectrum of 0 to 1800 nm (equivalent to 2.85λ). At an experimental incident angle of 85° within the interferometer, the detection of the segmented plane mirrors manifests a range of 2.85λ, aligning closely with theoretical and simulated expectations for piston error detection (specifically, 2.868λ).

**Figure 19:**
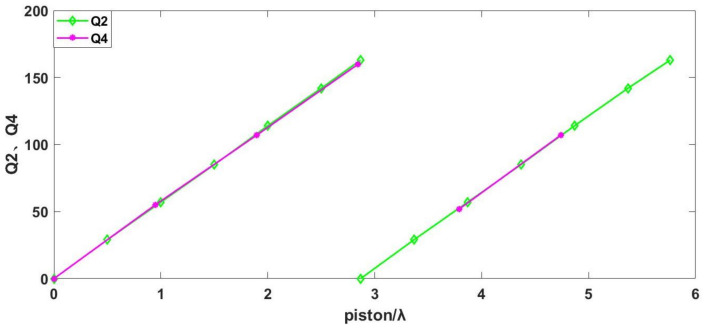
Schematic diagram of the relationship between Q2, Q4, and piston errors at 85° incidence.

The ratio between the travel distance of interference fringes to the spacing of fringes in the 85° incidence experiment is defined as Q4. A comparative analysis was conducted to assess the correlation between Q4 and the piston errors. This was then compared with the previously simulated 85° incidence experiment, specifically focusing on the relationship between Q2 and the piston errors, as illustrated in Figure 19.

The correlation observed in the 85° incidence experiment between Q4 and piston errors and the correlation between Q2 and piston errors in the simulation analysis are depicted in the figure above.

To sum up, in order to visualize the movement of interference fringes in experiments and simulations, this paper defines the ratio between the travel distance of interference fringes and the spacing of fringes as Q, and provides a graph of the relationship between Q and the change in piston errors. The simulation and experimental data are compared in Figure 16 and Figure 19, and the results show that the relationship between Q and the change in piston errors in the experiment is consistent with that in the simulation.

## 5. Discussion

In this paper, the author advocates the utilization of the grazing incidence interferometric detection technique for discerning the segmented plane mirrors, by adjusting the incident angle of the interferometer from 85° to 75°; with this method, the rough and precise adjustment of the segmented plane mirrors can be realized. This approach augments the interferometer’s detection scope in comparison to conventional interferometric detection methods. The application of θ as the incident angle during interferometric detection extends the scope of detection in the incident direction to $\frac{D}{\cos\theta }$ (where D represents the aperture of the interferometer). Consequently, this expansion encompasses the entirety of the sub-mirror column within the segmented plane mirrors. Moreover, in contrast to conventional interferometric detection techniques, the present method extends the detection range for piston errors. Specifically, elevating the incident angle to 89° facilitates a detection range extending up to 14.32λ.

At present, there are many methods to detect segmented plane mirrors. In contrast to the advanced grazing incidence interferometric detection method proposed in this study, the dispersion fringe method offers a straightforward optical path, user-friendly operational procedures, and an expansive measurement range. Nevertheless, its application is predominantly relegated to the initial rough co-phase stage due to inherent limitations in accuracy [15]. The Shack–Hartmann detection method, incorporating both wide and narrow bands, mandates the use of a mutual correlation algorithm, involving the computation of numerous feature points to resolve the shape of the segmented plane mirrors. Unfortunately, this approach demonstrates suboptimal detection efficiency [16]. The dual-wavelength or multi-wavelength co-phase detection method takes a lot of time to detect [17], and the multi-wavelength interferometer is expensive, and it is more suitable for detecting the primary mirror with curvature, rather than for segmented plane mirrors. Pentaprism scanning detection is susceptible to manufacturing errors in system components, positional variations induced by pentaprism movement, inherent autocollimator measurement errors, environmental vibrations, and temperature fluctuations. Consequently, this method lacks the requisite detection precision for segmented plane mirrors, exhibiting limitations in both accuracy and range, coupled with the issue of 2π blurring [18].

In contrast to alternative detection methods employed for segmented plane mirrors, the grazing incidence interferometric detection method demonstrates concurrent fulfillment of superior precision, heightened efficacy, and an expansive detection scope along the incidence direction. Moreover, this method effectively extends the detection range of segmented plane mirrors in relation to piston errors, thereby addressing the 2π ambiguity inherent in co-phase detection.

## 6. Conclusions

An innovative methodology tailored to the complex task of detecting piston errors within the framework of co-phase detection applied to segmented plane mirrors is introduced in this paper. The proposed technique, predicated upon grazing incidence interferometric detection, was rigorously substantiated through comprehensive simulation analyses and empirical experimentation. The outcomes underscore a discernible augmentation in the detection range for piston errors, expanding from 0.5λ to 2.868λ as the incident angle is elevated to 85°. Furthermore, judicious manipulation of the incident angle within the range of 85° to 75° affords nuanced control over the detection coverage, enabling a seamless transition from rough adjustments encompassing a broad scope to precise, small-scope sub-mirror detection. This method is characterized by its remarkable attributes of heightened precision, operational efficiency, and an extended detection scope concerning segmented plane mirrors along the incident direction. Critically, it adeptly addresses the inherent 2π ambiguity in co-phase detection. These findings contribute substantively to the field, providing an effective solution to the challenge of piston error detection in segmented plane mirrors, with potential implications for broader applications in large-aperture space optical imaging systems.

## Figures and Tables

**Table 1:** Advantages and disadvantages of contemporary segmented mirror detection methods.

Detection Methods	Coverage	Detection Range	Detection Accuracy	Detection Efficiency	2π Ambiguity
Broad- and narrow-band S-H detection	Large	Large	Comparatively high	Low	No
Dispersion fringe method	Large	Large	Low	Low	No
Pentaprism scanning detection	Large	Large	Low	Low	Yes
Dual-wavelength co-phase detection	Small	Comparatively large	Comparatively high	Low	No
Interference detection method	Small	Small	High	High	Yes

## Data Availability

The data are not publicly available due to secrecy.
